# Sleep apnea detection by a cardiac resynchronization device integrated thoracic impedance sensor: A validation study against the gold standard polysomnography

**DOI:** 10.1371/journal.pone.0195573

**Published:** 2018-04-06

**Authors:** Fabian Barbieri, Wolfgang Dichtl, Anna Heidbreder, Elisabeth Brandauer, Ambra Stefani, Agne Adukauskaite, Thomas Senoner, Wilfried Schgör, Florian Hintringer, Birgit Högl

**Affiliations:** 1 University Hospital for Internal Medicine III (Cardiology and Angiology), Medical University Innsbruck, Austria; 2 University Hospital for Neurology, Medical University Innsbruck, Austria; 3 Department of Neurology, Division of Sleep Medicine and Neuromuscular Disorders, University Hospital Münster, Germany; Kurume University School of Medicine, JAPAN

## Abstract

**Background:**

Sleep disordered breathing is a common but often undiagnosed comorbidity in heart failure patients. Cardiac implantable electronic devices used for cardiac resynchronization therapy (CRT) may detect sleep apnea by use of a transthoracic impedance sensor. Validation of the AP scan® algorithm (Boston Scientific®) was performed by using the diagnostic gold standard polysomnography (PSG).

**Methods:**

Forty-one patients with impaired left ventricular ejection fraction, frequent right ventricular pacing due to atrioventricular block and heart failure symptoms despite optimal medical therapy underwent upgrading to biventricular pacing. Within one month after left ventricular lead implantation, sleep apnea was assessed by single-night PSG and AP scan® measurements.

**Results:**

AP scan® measurements were valid in only 21 of 41 (51.2%) patients in the index night of the PSG. The PSG determined apnea-hypopnea index did not correlate statistically significant with the AP scan® measurements (r = 0.41, 95% confidence interval -0.05–0.72, p = 0.07). The degree of overestimation is displayed by using the Bland-Altman method: mean difference -12.4, standard deviation ± 15.8, 95% confidence interval -43.3–18.6.

**Conclusions:**

In heart failure patients receiving CRT upgrading, the AP scan® algorithm may need further improvement before it can be recommended for sleep apnea detection.

## Introduction

Cardiac resynchronisation therapy (CRT)–also known as biventricular pacing—is an established treatment option for heart failure patients with electromechanical dyssynchrony shown by a wide QRS complex [1]. Besides its classical indication due to a left bundle branch block (which is found in about one quarter of patients with a systolic ejection fraction below 35%), it is also beneficial in patients suffering from pacing-induced cardiomyopathy. This form of heart failure develops in some pacemaker patients who need chronic right apical ventricular stimulation because of high-degree atrioventricular block. Right apical ventricular pacing results in iatrogenic left ventricular dyssynchrony similar to what is seen in patients with a left bundle branch block. This dyssynchrony can be effectively treated by additional implantation of a left ventricular lead, which results in biventricular instead of right apical electrical activation of the heart. This procedure is referred as “CRT upgrading” [2].

Sleep-disordered breathing (SDB) is common in patients with cardiovascular diseases, and is a marker of poor clinical outcome [3]. SDB is prevalent in up to 80% in patients with heart failure [4]. Especially central sleep apnea (CSA) and Cheyne-Stokes respiration are common in congestive heart failure [5]. Respiratory events are distinguished between apnea (cessation of breathing for ≥ 10 seconds) and hypopnea (≥30% reduction of airway flow signal). They are differentiated into central, obstructive, and mixed (which is mostly central in the beginning and turns to an obstructive apnea in the end) respiratory events. Central sleep apnea with Cheyne Stokes respiration is characterized by recurrent central apneas or central hypopneas alternating with respiratory phase exhibiting crescendo-decrescendo pattern of flow with a typical cycle length of 45–60 seconds [6]. To make a reliable diagnosis of SDB in patient with HF, a polygraphy or polysomnography (PSG) is needed. As these examinations are time consuming and expensive, easier and less cost-intensive screening tools are needed.

Minute ventilation sensors of cardiac implantable electronic devices (CIED) measure ventilation by means of thoracic impedance changes between the case and the electrode tip. Primarily used to adjust pacing rate during exercise, these sensors can also be used to detect respiratory events during predefined sleeping hours, as transthoracic impedance is increased during inspiration and decreased during expiration. Recently, new algorithms have been developed in CIED to screen for sleep apnea. In the DREAM study [7], Defaye et al. showed that the respiratory events detected by the SAM® (sleep apnea monitoring) algorithm available in Livanova® (formerly SORIN®) pacemakers correlates well with the apnea-hypopnea index (AHI) assessed by the gold standard PSG in a cohort of 40 patients.

A second algorithm for the detection of SDB called AP scan® is used in CIED from another vendor (Boston Scientific®). It has recently been suggested in a clinical study as a risk indicator for new-onset atrial fibrillation [8]. On the programmer screen, a threshold line of the AP scan® is drawn at 32 events per sleeping hour to detect severe sleep apnea. This threshold is based on an internal retrospective analysis of the company from pacemaker respiration data for nights with simultaneous PSG, by using a receiver operating characteristic (ROC) analysis for the algorithm detecting a PSG-AHI ≥ 30.

A first study to detect sleep disordered breathing by using the herein evaluated thoracic impedance sensor in pacemakers (at time, produced by a company called Guidant®) has already been performed in 2006: Shalaby et al. [9] enrolled sixty patients, of whom severe sleep apnea (PSG-AHI ≥ 30) was diagnosed in 32 patients (only 1% central sleep apnea events). The pacemaker derived information about pathological respiratory events during sleep correlated well with the AHI of the PSG (r = 0.80, p < 0.01), and the algorithm identified patients with severe sleep apnea with 82% sensitivity and 88% specificity.

In this study, we set out to further validate the AP scan® algorithm by using single-night PSG examinations in heart failure patients receiving CRT upgrading, as part of the ongoing UPGRADE study.

## Materials and methods

### Study design

The UPGRADE study (ClinicalTrials.gov Identifier: NCT01970423) is an ongoing investigator-driven prospective assessment of CRT upgrading in sleep apnea. Inclusion criteria are heart failure symptoms despite optimized neurohumoral medical therapy (New York Heart Failure Association Class II, III or ambulatory IV), left ventricular ejection fraction below 40% and the need of right ventricular pacing above 40% despite optimal device programming. Exclusion criteria are end-stage heart failure, a glomerular filtration rate below 30 ml/min/1.73 m^2^, a life expectancy below one year, women with childbearing potential, drug abuse, hyperthyreosis, and contrast allergy. The study protocol was approved by the ethics committee of the Medical University Innsbruck. All patients gave informed consent.

### Polysomnography

PSG was performed and analysed according to standard procedure of the American Academy of Sleep Medicine and included six electroencephalography derivations, horizontal and vertical electrooculography, mental and submental electromyography, oronasal pressure cannula, thermistor, thoracic and abdominal respiratory movements (piezo) and transcutaneous oxygen saturation. In accordance with recommendations of the SINBAR group [10], all PSG included surface electromyography of both upper (flexor digitorum superficialis) and lower (tibialis anterior muscles) extremities. Time in bed was 8 hours (usually, 22 to 6 o´clock). The apnea-hypopnea index is defined as the sum score of all respiratory events including obstructive, central and mixed apneas and hyponeas per hour of sleep.

### AP scan® sensor

The AP scan® sensor measures fluctuations in transthoracic electrical impedance (assessed in ohms) that occur with inspiration and expiration. It uses filters to limit the frequency range of the fluctuations with a lower limit of about 6 breaths/minute to an upper limit that varies with heart rate from about 29 breaths/min to about 70 breaths/min. This filtering largely eliminates fluctuations due to activity and cardiac stroke. What remains is a respiratory waveform. The algorithm uses this waveform to detect breaths and calculate relative tidal volumes for each detected breath. By running averages of tidal volumes, an apnea/hypopnea event is defined as a period of 10 seconds or longer during which the tidal volumes remain below 74% of the running average, and/or breaths are not detected. The AP scan® sensor is not sensitive enough to distinguish between apneas and hypopneas, as it displays the number of occurrences of either one per hour of sleep and therefore should correlate very well to the PSG derived AHI.

Sleep time for AP scan® detection was programmed in the device according to patients habits, in most cases between 23 and 6 o´clock. The value given by AP scan® for that timeframe was then compared to the AHI measured by the PSG.

1. In the first step AP scan® data were compared to the AHI obtained during the whole PSG index night.
2. In a second step AP scan® data were compared to PSG data derived only for the programmed timeframe.
3. In a third step mean AP scan® data from the first 5 to 30 nights after implantation were compared to the results of PSG.
4. Finally, the total amount of AP scan® data available during long-term follow up was assessed as it actually yielded such measurements much less than expected.

### Statistics

Continuous variables are expressed as median and interquartile range, categorical variables are reported as number and percentage. Differences of categorical variables were compared by using the chi-square test. Intermodality correlations were calculated by using bivariate spearman correlation and for analysis of differences, Bland-Altman method was performed. Therefore Kolmogorov-Smirnov tests were utilized to verify normal distribution of differences. Statistical analysis was conducted by using IBM SPSS, version 24 (IBM Corporation, Armonk, NY, USA), graphics were designed by using GraphPad PRISM, version 5 (GraphPad Software, Inc., La Jolla, CA, USA). P-values < 0.05 were considered as statistically significant.

## Results

### Subjects

Between January 2014 and March 2017, forty-one patients were recruited at the Medical University Innsbruck, Austria: age was 75.0 (73.0–77.5) years; 75.6% were men; 43.9% suffered from ischemic cardiomyopathy; left ventricular ejection fraction was 28.0% (21.9–36.0); 58.5% suffered from chronic atrial fibrillation; body mass index was 25.7 kg/m^2^ (23.9–28.5). CRT-D devices (Boston Scientific® INCEPTA® or AUTOGEN®) were used in 18 (43.9%) patients; CRT-P devices (Boston Scientific INLIVEN® or VISIONIST®) were used in 23 (56.1%) patients.

### Characteristics of sleep obtained by PSG

All 41 patients underwent PSG for one night. Median time in bed (TIB) was 480 minutes (459.5–522.5), total sleep time (TST) was 349 minutes (282.0–417.0), and sleep period time (SPT) was 459 minutes (425.0–509.5) with sleep stage N1: 20.3%/ SPT (14.8–25.5), N2: 37.5%/ SPT (28.6–47.6), N3: 2.9%/ SPT (0.0–7.9), rapid eye movement (REM): 8.2%/ SPT (4.1–16.0), wake after sleep onset (WASO) 23.4%/ SPT (14.9–38.4). Mean sleep efficiency (% sleep period time [SPT]) was 73.9% (56.9–81.8). The mean sleep latency was 13.3 minutes (4.4–43.3).

### Prevalence of sleep-disordered breathing in patients undergoing CRT upgrading

**Fig 1:**
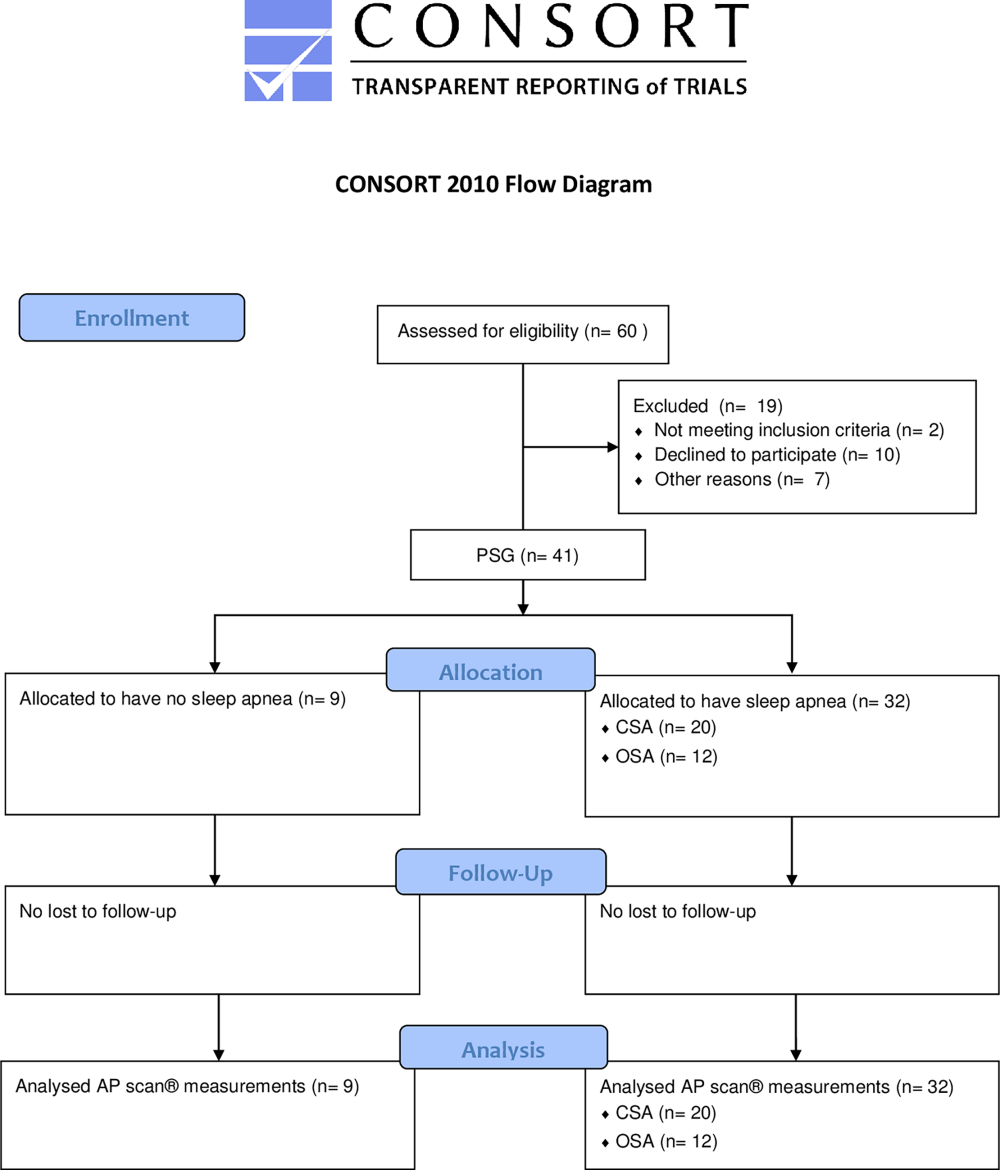
CONSORT diagram of the UPGRADE study.

As shown in Table 1 and Fig 1, nine patients (22.0%) had no sleep disordered breathing, 20 patients (48.8%) were diagnosed having central sleep apnea (CSA) (mild: n = 4, moderate: n = 6, severe: n = 10), and 12 patients (29.3%) suffered from obstructive sleep apnea (OSA) (mild: n = 6, moderate: n = 4, severe: n = 2).

**Table 1:** Baseline charcteristics of the study cohort and sleep disordered breathing evaluated by single-night PSG.

	n / median	% / IQR
Age (years)	75	73.0–77.5
Gender		
female	10	24.4%
male	31	75.6%
BMI (kg/m^2^)	25.7	23.9–28.5
CRT		
CRT-P	23	56.1%
CRT-D	18	43.9%
NYHA		
II	8	19.5%
III	30	73.2%
Ambulatory IV	3	7.3%
COPD	7	17.1%
LVEF (%)	28.0	21.9–36.0
LVEDV (ml)	168.0	137.5–211.5
LVEDD (mm)	67.0	57.0–71.0
SA		
None	9	22.0%
CSA	20	48.8%
OSA	12	29.3%
Sleep efficiency (% SPT)	73.9	56.9–81.8
TIB (min)	480.0	459.5–522.5
TST (min)	349.0	282.0–417.0
Total wake time (min)	104.0	56.0–166.8
Sleep onset latency (min)	13.3	4.4–43.3
REM sleep latency (min)	121.0	59.0–261.0
SPT (min)	459.0	425.0–509.5
N1 (% SPT)	20.3	14.8–25.5
N2 (% SPT)	37.5	28.6–47.6
N3 (% SPT)	2.9	0.0–7.9
REM (% SPT)	8.2	4.1–16.0
WASO (% SPT)	23.4	14.9–38.4
AHI (events per hour of sleep)	19.4	6.0–34.3
ODI > 4%	15.5	4.2–34.2
O2 saturation baseline	95.0	93.1–96.6
Amount waking phasis	27.0	19.0–49.0
Total duration central apnea (min)	2.6	0.4–45.0
Average duration central apnea (sec)	17.3	14.4–22.6
Total duration obstructive apnea (min)	0.6	0.0–2.5
Average duration obstructive apnea (sec)	16.2	0.0–25.0

### Characteristics of AP scan® measurements during the index night of the PSG

During the index night of PSG only 21 patients (51.2%) had a valid AP scan® result (no SA: n = 5 [55.6%], CSA: n = 10 [50.0%], OSA: n = 6 [50.0%]; p = 0.96).

### Correlation of the AP scan® measurements with the AHI during single night PSG

**Fig 2:**
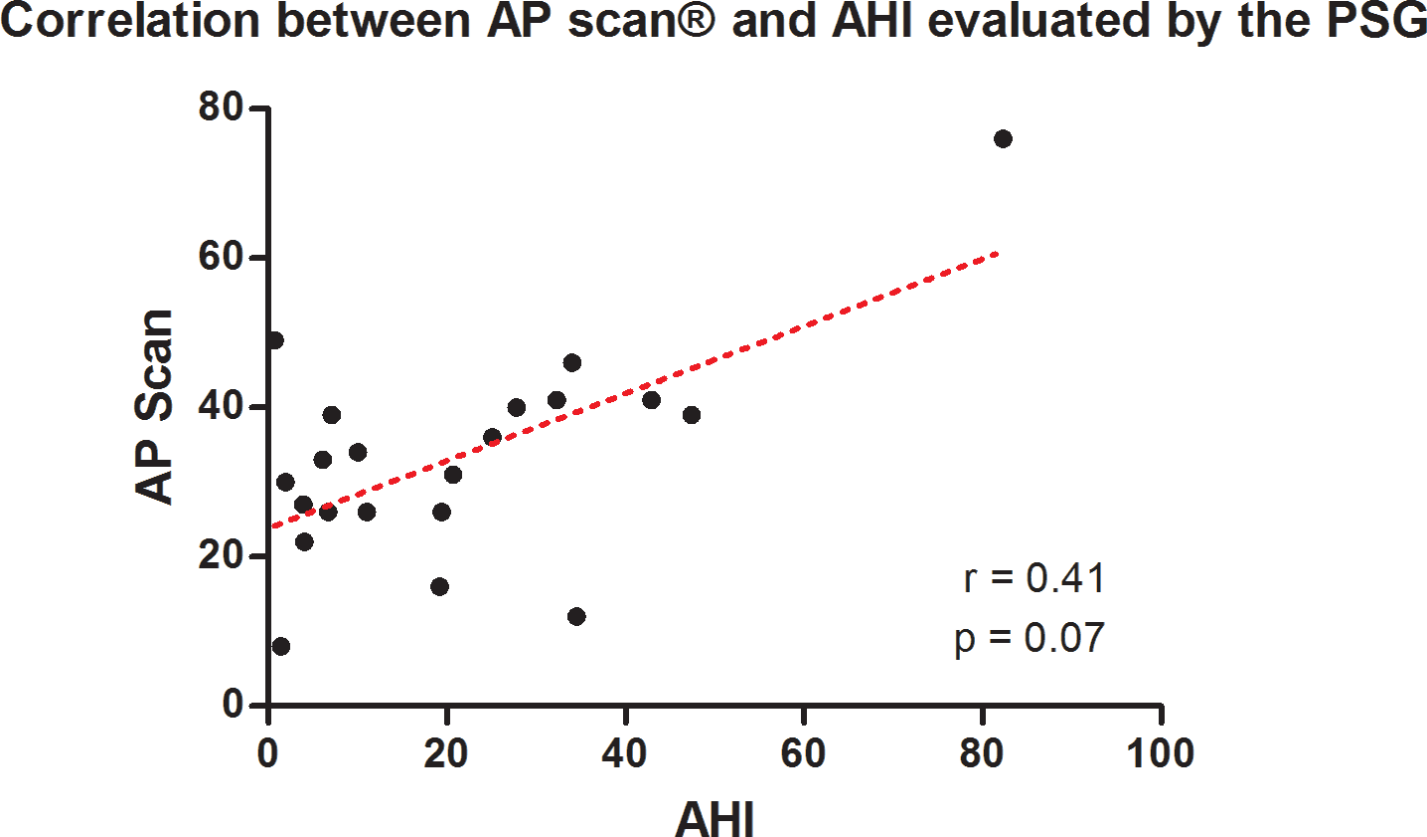
Scatter plot between AP scan® measurements and the AHI evaluated by PSG (in events/h).

**Fig 3:**
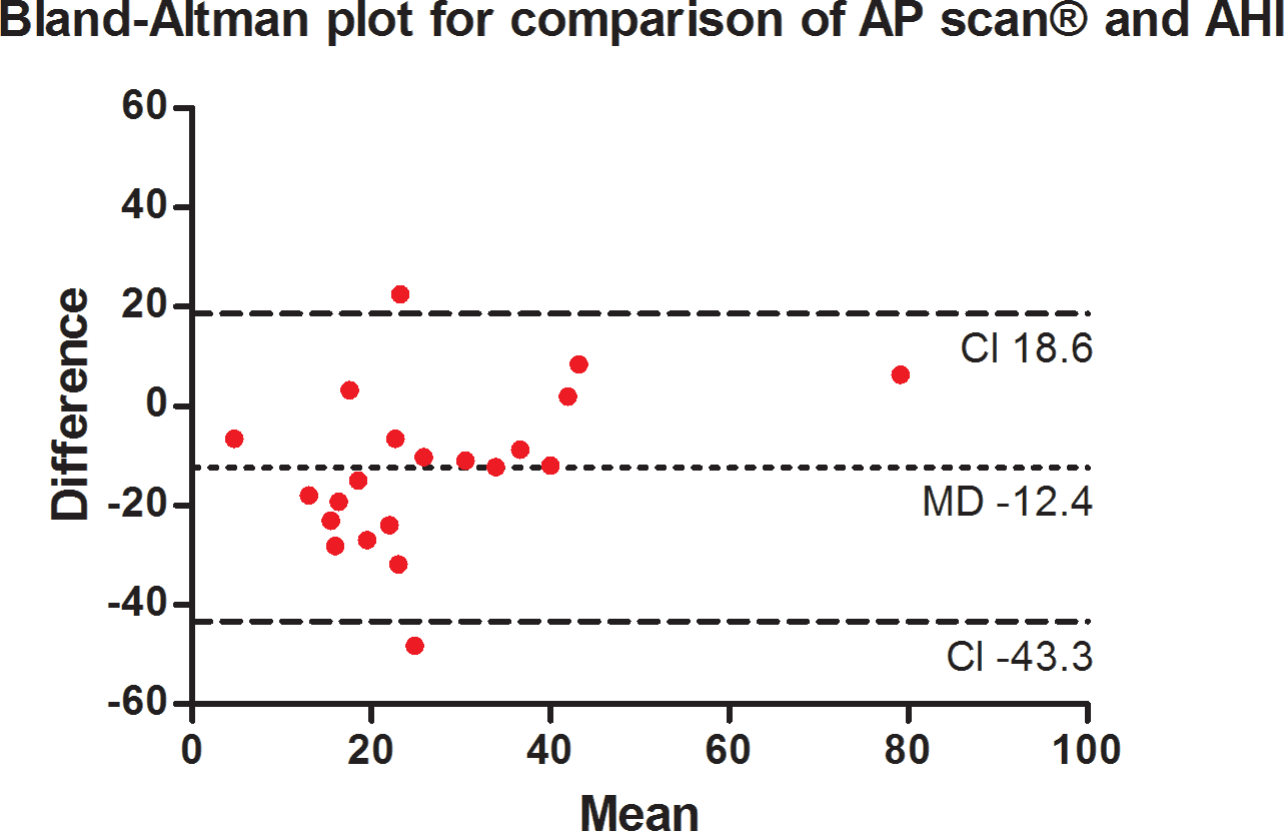
Bland-Altman plot (y axis: Difference between PSG-AHI and AP scan® measurements in the index night; x axis: Mean of the AP scan® and PSG-AHI), showing mean of the differences (bias) presented with the 95% confidence interval.

**Fig 4:**
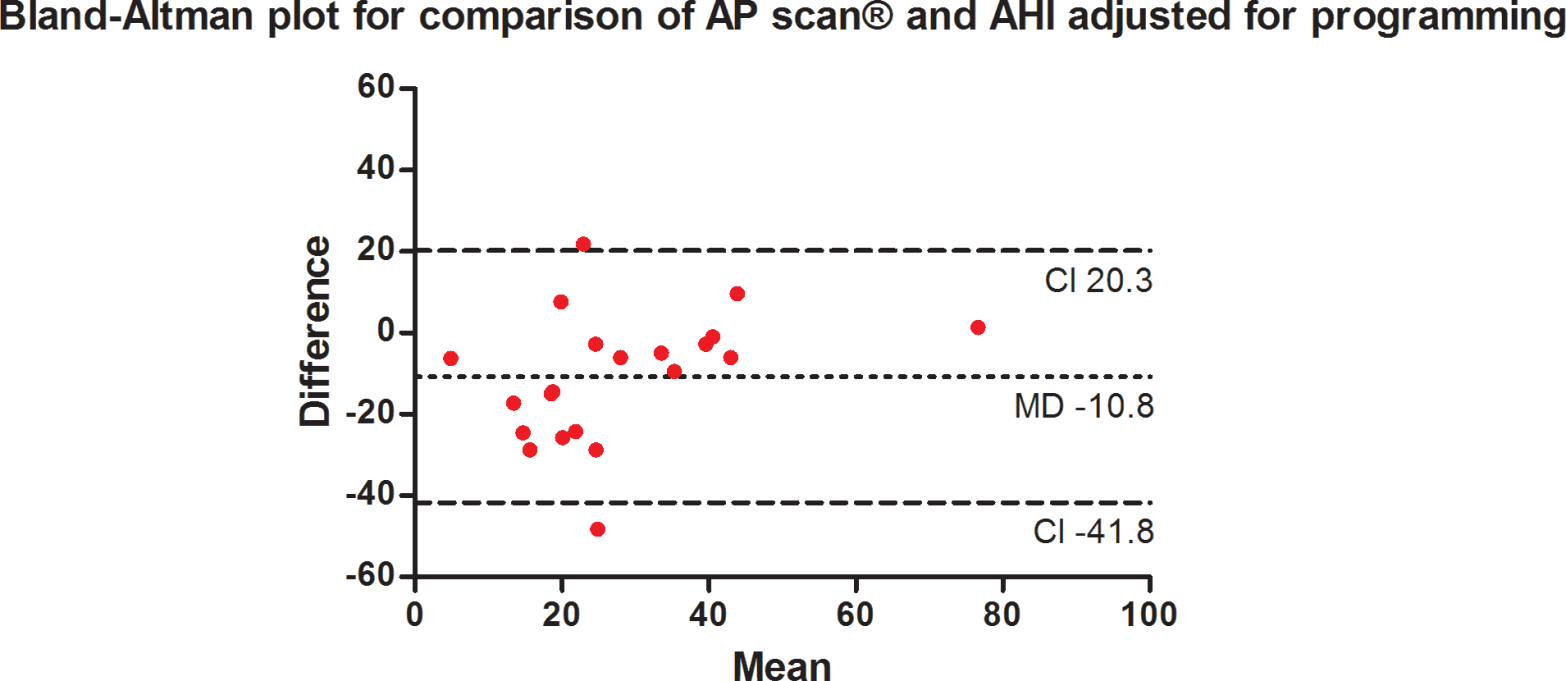
Bland-Altman plot (y axis: Difference between PSG-AHI adjusted to the night hours programmed and AP scan® measurements in the index night; x axis: Mean of the AP scan® and PSG-AHI), showing mean of the differences (bias) presented with the 95% confidence interval.

As shown in Fig 2, AP scan® measurements showed only a poor correlation to the AHI measured by PSG (r = 0.41, 95% confidence interval (CI) -0.05–0.72, p = 0.07). The degree of inequality is therefore demonstrated by the Bland-Altman method (Fig 3; mean difference -12.4, standard deviation ± 15.8, 95% CI -43.3–18.6) illustrating a mean overestimation of 12.4 by the AP scan® and the varying spectrum of measurements displayed by the confidence interval. The comparison of AHI adjusted for the night hours of AP scan® programming and AP scan® measurements revealed similar correlation (r = 0.41, 95% CI -0.04–0.72; p = 0.06) and overestimation (Fig 4; mean difference -10.8, standard deviation ± 15.8, 95% CI -41.8–20.3).

### Sensitivity and specificity of the AP scan® measurements compared with the PSG-AHI in severe SA

The evaluation of sensitivity and specificity was performed by using a threshold AP scan® value of 32/h (n = 11, 52.4%), which is intended to correlate well to a clinical threshold for severe SA in the PSG index night (AHI ≥ 30/h; n = 6, 28.6%), according the information given by the company. Analysis of 21 patients with successful AP scan® measurement resulted in a sensitivity of 83.3% (false negative rate / type 2 error = 16.7%), specificity was 60.0% (false positive rate / type 1 error = 40.0%).

### Sensitivity and specificity of mean AP scan® measurements compared with primary PSG diagnosis

Only small differences were found when using a mean value for AP scan® measurements for the nights 5–30 post implantation. Sensitivity resulted in 80.0% (false negative rate / type 2 error = 20.0%), specificity was 60.0% (false positive rate / type 1 error = 40.0%).

### Availability of AP scan® measurements during long-term follow up

**Fig 5:**
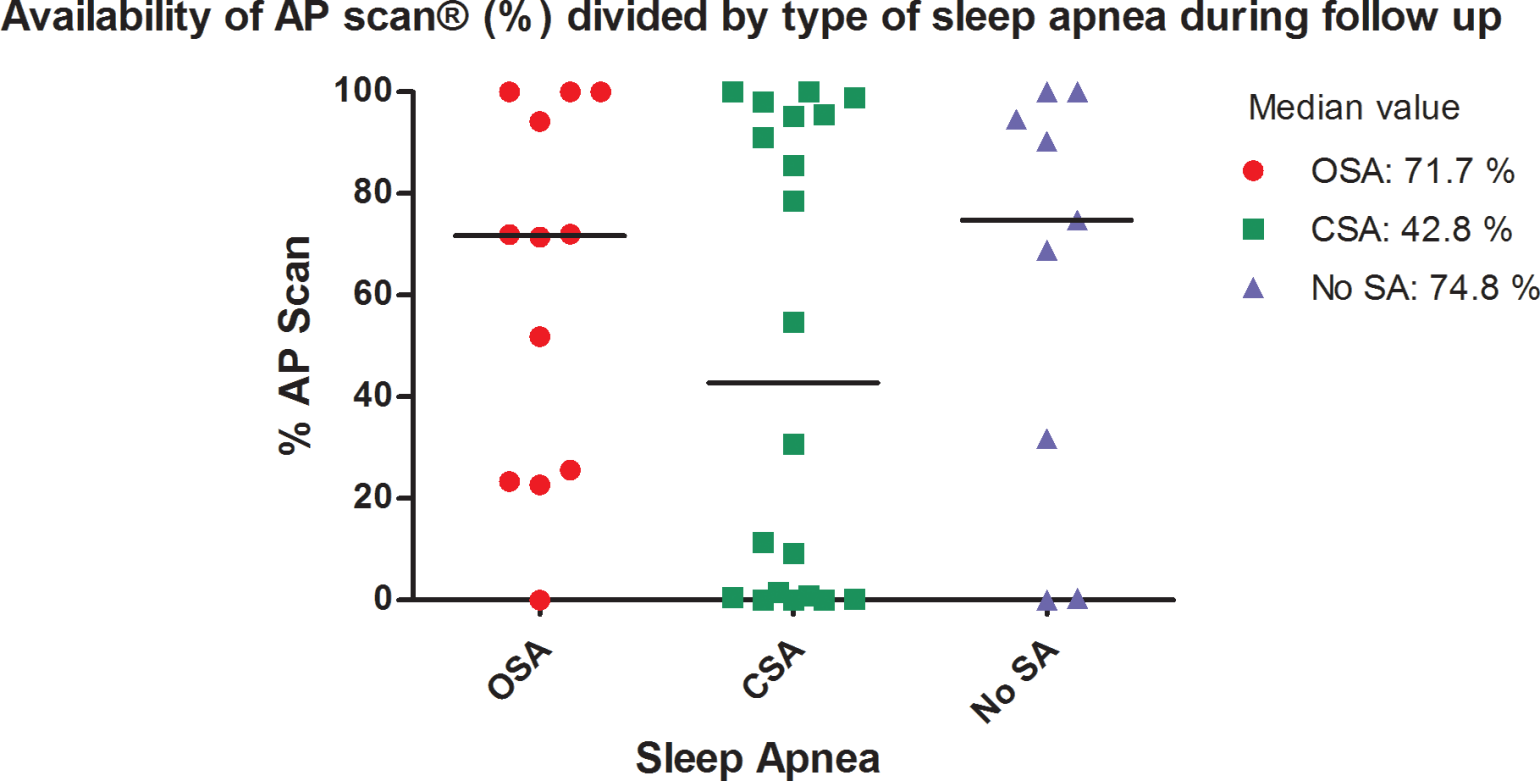
Grouped scatter plot showing the availability of AP scan® measurements (percentages of nights analysed) during long-term follow up, stratified to the subgroups OSA, CSA and absence of SA.

Overall, valid AP scan® measurements were obtained in only 52.6% of all nights analysed (n = 4061 out of 7721). In Fig 5, the median of percentages of AP scans® available in each study patient are shown according to the three subgroups of sleep apnea diagnosed in the PSG: no significant SA (median = 74.8%, 16.1–97.3), CSA (42.8%, 0.7–95. 6) and OSA (71.7%, 24.0–98.6).

## Discussion

More than a decade ago, studies introduced the fundamental possibility to detect sleep-related breathing disorders by a transthoracic impedance sensor in pacemakers [9,11,12]. In the meantime such algorithms to detect sleep apnea have become routinely available by two vendors (SAM® by Livanova® and AP scan® by Boston Scientific®, respectively). The reasons why such a screening tool in CIED patients is valuable are obvious: SDB events are markers for adverse prognosis [3–5,13,14] and can be treated easily by using PAP therapy or other therapeutic options including surgery. Secondly, performing cardiorespiratory polygraphy or full PSG is a time- and cost-consuming strategy for screening.

In this study, we report two important observations: (1) The correlation between the AP scan® measurement and the AHI on a single-night PSG is not optimal. The AP scan® measurement commonly overestimates sleep apnea and needs improvement before it can be accepted as a screening tool in clinical practice. (2) Almost half of the nights analysed did not yield an AP scan® measurement, and the failure to yield AP scan® measurements was apparently more common in patients suffering from CSA.

The transthoracic impedance based respiratory sensor was originally designed to measure respiration during activity and to adapt the cardiac pacing rate based on the patient’s minute ventilation. It works well for that purpose because the respiratory fluctuations are obviously larger during activity then they are when the patient is sleeping. When patients are sleeping, the sensor often operates at the lower limit of its sensitivity, and in some patients, the signal is just too weak to reliably measure respiratory events. The algorithm continually checks the amplitude of the respiratory impedance fluctuations and if they get too small, it will suspend breath detections to avoid erroneous results. If there are less than two hours in a given night where the respiratory signal is strong enough to reliably sense, then no AP scan® value will be reported for that night. These amplitudes are highly patient-specific, so there are some patients whose amplitudes are rarely large enough for more than two hours a night, and for those patients one rarely gets an AP scan® value on the trend graph.

In a recent study by Moubarak et al. analysing dual-chamber Livanova® Reply 200DR or Kora 100DR pacemakers in 65 patients [15], only 7 patients (11%) had more than 10% invalid nights. In the other 58 patients, SAM® data could be measured in 98% of the nights. Similar findings were found in a study by Dias et al. [16] analysing 60 patients with DDDR Reply 200DR pacemakers in whom SAM® data for the PSG night was available in 90%. This is in strong contrast to the here presented UPGRADE study, where AP scan data® could be retrieved in only about half of the nights analysed. This discrepancy was probably due to the high percentage of patients with CSA, in whom valid AP scan® measurements were particularly low (42.8%). Unfortunately, the device does not record why the respiration signal was deemed invalid.

The overall performance of the AP scan® sensor was worse than expected when validated against a single night AHI measurement by the gold standard PSG. In comparison the study by Shalaby et al. [9] showed a better correlation between the calculated respiratory events by Boston Scientific® (formerly Guidant®) pacemakers and the AHI measured by using PSG (r = 0.80). Notably, obstructive sleep apnea was the predominant type of sleep disordered breathing in this trial, and only 1% of all SDB episodes were central sleep apnea. Nevertheless, the reasons for this discrepancy remain unclear, and necessitate further studies and/or technical improvements on this technology.

## Conclusion

UPGRADE is the first study analysing the new Boston Scientific® algorithm AP scan® for SDB events detection in CRT patients. At least in this cohort of patients suffering from advanced heart failure, this technology may need further improvement before it can be recommended for sleep apnea detection in clinical routine.

## Supporting information

S1 File TREND statement checklist. (PDF)

S2 File UPGRADE clinical trial plan. (PDF)

## Abbreviations

AHI: apnea-hypnea index
CIED: cardiac implantable electronic device
CRT: cardiac resynchronization therapy
CSA: central sleep apnea
OSA: obstructive sleep apnea
PSG: polysomnography
SDB: sleep-disordered breathing
