# Supplementary material for: Sleep apnea detection by a cardiac resynchronization device integrated thoracic impedance sensor: A validation study against the gold standard polysomnography
Source: PLoS One. 2018 Apr 6;13(4):e0195573. doi: 10.1371/journal.pone.0195573 (PMC5889179; doi:10.1371/journal.pone.0195573)
Supplement: S2 File — (PDF) [file pone.0195573.s002.pdf]

UPGRADE

Sleep Apnea and New-Onset Cardiac Resynchronisation in Patients With Conventional Right Ventricular Pacing – A Randomized Clinical Trial

Grant applicant / Principal Investigator

Priv.-Doz. DDr. Wolfgang Dichtl

Phone: 0043 512 504 81388
Fax: 0043 512 504 22767
E-mail: wolfgang.dichtl@uki.at

Head of the department

Univ.-Prof. Dr. Wolfgang Michael Franz

Department of Internal Medicine III / Cardiology and Angiology
Innsbruck Medical University
Anichstr. 35, A-6020 Innsbruck

Innsbruck, 28.10.2013

1 SUMMARY

Within the last decade cardiac resynchronization therapy (CRT) has been proven to be an effective therapy to reduce morbidity and mortality in chronic heart failure patients with wide QRS complex, in particular complete left bundle branch block [1-8]. New indications have recently been established, including patients with mild symptoms [9] and patients in need of conventional pacing such as high-grade atrioventricular block [10, 11].

More than half - up to 80% [12] - of patients with heart failure suffer from concomitant sleep apnea (SA), which further worsens symptoms and prognosis. Cardiac resynchronization therapy may ameliorate sleep apnea, but only the central form of sleep apnea (CSA). However, only very small uncontrolled studies with mainly less than 20 patients have been reported so far concerning the interactions between CRT and sleep apnea, and no data are available in patients with conventional right ventricular pacing undergoing upgrading to CRT [13].

Therefore, we want to perform a study called UPGRADE which is characterized

- being the first randomized study comparing the effects of new-onset cardiac resynchronization therapy on moderate and severe sleep apnea, defined by an respiratory disturbance index (RDI) of ≥ 15/h; polysomnography is used to exclude patients with obstructive sleep apnea

- being the first trial in patients with conventional right ventricular pacing which is known to decrease cardiac function, induce heart failure and atrial fibrillation [14]

- using a new technology called AP Scan® (described below) which enables continuous and reliable monitoring of sleep-disordered breathing (SDB); this technology is further validated with polysomnography, the gold standard in the diagnosis and follow-up in patients with sleep apnea

The minute-ventilation sensor has been used for years for optimal physiologic pacing rate adaption in patients with pacemakers and chronotropic incompetence. This sensor now enables screening and follow-up of sleep-disordered breathing. It is expected that all conventional pacemakers will be able to analyze sleep apnea in the near future. This will substantially increase the number of diagnosed patients as

- 75% of all patients with severe sleep apnea are still not diagnosed [(15)]
- 60% of all patients with cardiac devices suffer from sleep apnea [(16-18)]

Unfortunately, one third of patients still do not benefit from CRT (so-called *non-responders*). On the other hand, up to 20% of patients greatly benefit and completely recover in terms of normalization of left ventricular ejection fraction and/or functional capacity (so-called *super-responders*). Research is urgently needed to decrease the number of non-responders and increase the number of super-responders.

Patient selection is still based on QRS duration and its morphology. Echocardiography and other imaging techniques for mechanical dyssynchrony assessment have failed to be a useful predictor for adequate patient selection [(19)]. Therefore, we further want to test whether CRT itself does not only improve concomitant sleep apnea, but also if preexisting sleep apnea predicts the response to CRT in patients with previously conventional right-ventricular pacing undergoing an upgrade to CRT by additional implantation of a left ventricular lead.

2 STUDY DESIGN

The study is a multicenter randomized clinical trial with a cross-over design.

2.1 Aim of the study

primary study aim

- to analyse the interactions between sleep apnea and upgrading to cardiac resynchronisation therapy in patients with reduced left ventricular ejection fraction and conventional right ventricular pacing due to AV block or atrial fibrillation with slow ventricular conduction (including patients after total AV node ablation):

patients with advanced sleep apnea, defined by a mean respiratory disturbance index (RDI) ≥ 15/h as detected by the CRT-P device INLIVEN® or CRT-D device INCEPTA® (both from Boston Scientific®) in a run-in phase with the newly implanted LV lead still being inactivated, are further assessed by polysomnography; patients with central sleep apnea are randomised to CRT versus continuous conventional right ventricular pacing for three to five months; possible effects on sleep apnea are assessed by the device and follow-up polysomnography. Afterwards, there will be a cross-over to the other treatment arm for another three to five months.

secondary study aims

- the cardiac response to CRT is correlated to preexisting sleep apnea

- the RDI as assessed by the CRT-P device INLIVEN® or CRT-D device INCEPTA® (both from Boston Scientific®) with the AP Scan® is correlated with the gold standard polysomnography

2.2 Endpoints

primary endpoint = improvement of moderate / severe central sleep apnea (RDI ≥ 15/min) due to new onset CRT as compared to ongoing conventional right ventricular pacing

- reduction of mean RDI (respiratory disturbance index) as assessed by AP Scan® in the first 90-150 days after initiation to CRT as compared to conventionel RV pacing

- reduction of AHI (apnea-hypopnea index) as assessed by polysomnography within 90-150 days after initiation to CRT as compared to conventionel RV pacing

secondary endpoints = CRT response according to pre-existing sleep apnea (RDI 0-14/min versus ≥ 15/min)

- improvement of left ventricular ejection fraction and reduction in left ventricular endsystolic volume as assessed by transthoracic echocardiography

- decrease in NTproBNP / BNP plasma concentration

2.3 Flowchart

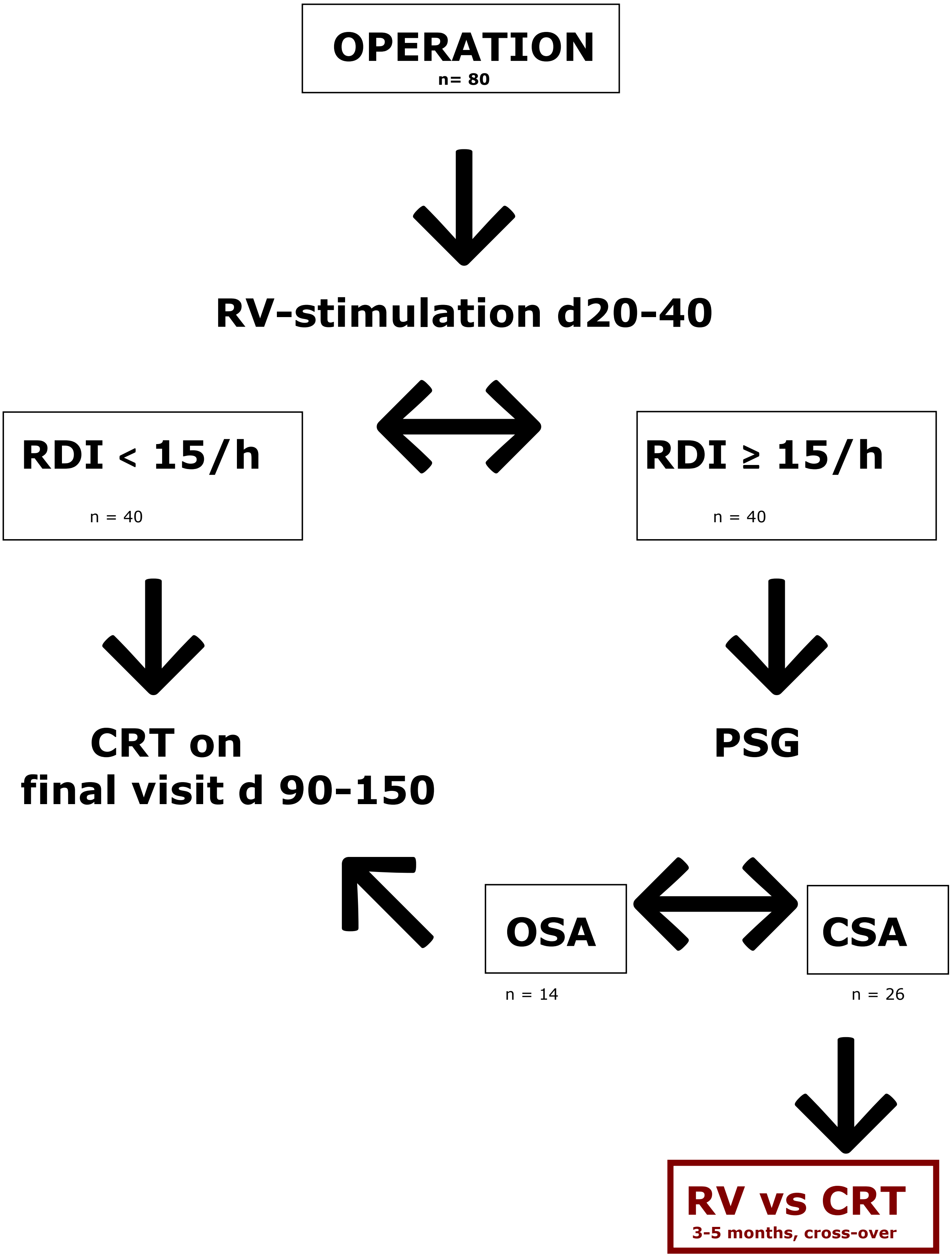

2.4 Patients

inclusion criteria

- left ventricular ejection fraction (assessed by echocardiography, CMR or LV laevography) < 50%
- implanted conventional pacemaker or ICD with a right ventricular pacing rate > 40% or planned „ablate and pace" therapy
- age 40 – 85 years

exclusion criteria

- terminal heart failure (NYHA IV)
- estimated glomerular filtration rate < 30 ml/min/1.73m^2
- premenopausal women
- life expectancy less than one year
- drug abuse
- incapability to understand the content of the study
- hyperthyreosis
- allergy to contrast medium
- inclusion in another clinical trial

VISIT 1 (all patients) **recruitment 1-30 days before CRT upgrade**

Implantation: CRTP INLIVEN® in patients LVEF between 35 und 50% *

CRTD INCEPTA® in patients with LVEF < 35% or established ICD indication

LV lead is still not activated

**ICD programming**	**VF-zone**	**> 240 ms (250/min)**	**2.5 sec**
	**VT-zone**	**> 320 ms (180/min)**	**60 sec**

*** it is also allowed to upgrade from conventional pacemakers to CRT-P in patients with LVEF < 35% according to patients preference and physicians discretion**

VISIT 2 (all patients) **30 ± 10 days postoperatively ± AV node ablation**
LV lead not activated

Device telemetry

- **if mean RDI 0-14/h: activation of the LV lead and initiation of CRT**
- **if mean RDI ≥ 15/h: LV lead remains inactivated, polysomnography**

ECG
TTE
Laboratory measurements
Organization of follow up visits

VISIT 3 (in patients with a baseline mean RDI ≥ 15/h), 50 ± 30 days postoperatively ± AV node ablation, LV lead still not activated

Polysomnography I

VISIT 4 (in patients with a baseline mean RDI ≥ 15/h and a central form of SA in PSG), 50 ± 30 days postoperatively ± AV node ablation, LV lead still not activated

RANDOMISATION

VISIT 5 (in randomised patients with a baseline mean RDI ≥ 15/h and a central form of SA in PSG), 90 ± 30 days after randomisation

Polysomnography II

VISIT 6 (in randomised patients with a baseline mean RDI ≥ 15/h and a central form of SA in PSG), 90 ± 30 days after randomisation

TTE
Laboratory measurements
Device telemetry
ECG

afterwards

CROSS-OVER

VISITE 7 (randomised patients) 120 ± 30 days after cross-over

Polysomnography III

VISIT 8A (all randomised patients), 120 ± 30 days after cross-over

TTE
Laboratory measurements
Device telemetry
ECG

afterwards
→ additional treatment if significant sleep apnea is still present
→ all patients are programmed to CRT

VISIT 8B (all non-randomised patients), 120 ± 30 days after CRT activation

TTE
Laboratory measurements
Device telemetry
ECG

afterwards
→ additional treatment if significant sleep apnea is still present

3 DETAILS OF THE PLANNED STUDY

3.1 Implantation

Implantation will be performed by experienced electrophysiologists / surgeons. Preoperative screening for vena subclavia occlusion / stenosis due to previously implanted pacemaker leads (e.g. by ultrasound sonography) is recommended.

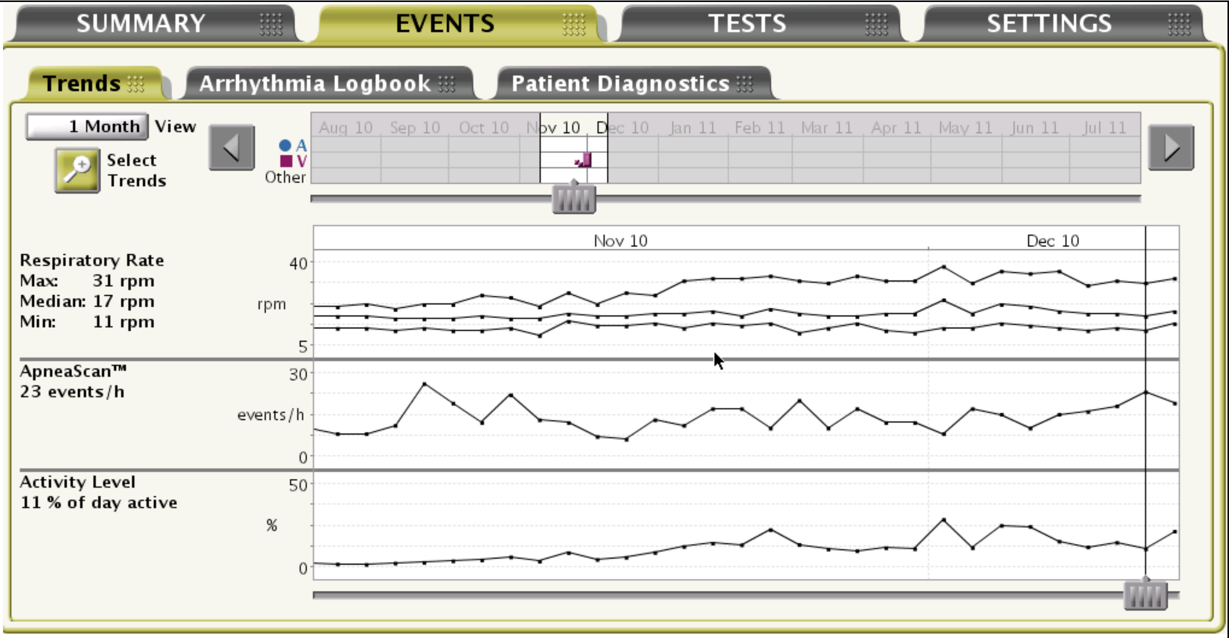

Diagram analyzing respiratory rate, AP Scan® and activity level. The AP Scan® (RDI) correlates with the clinical AHI (R=0,8; 82% sensitivity, 88% specifity, 88% PPV) to predict severe sleep apnea (AHI ≥30) [20].

3.2 Device Follow-up

Device follow-up will be performed at intervals specified by the protocol. AV interval optimization (in patients with SR) is performed by a validated device-specific algorithm (Smart Delay®) or by echocardiography according to the physicians discretion. The use of the device-specific chronotropic physiologic sensor (Right Rate®) is encouraged in all

patients, with an upper rate limit of approximately 200 – age (minimum 120/min, maximum 160/min). Lower pacing rate in patients with atrial fibrillation is set to 70 beats per minute. In case of CRT-D implantation, ICD programming is based on results of the MADIT RIT trial.

3.3 Polysomnography

Nocturnal polysomnography will be performed with a digital polygraph and will consist of vertical and horizontal electrooculography, surface electromyography according to the recommended SINBAR RBD montage, electrocardiography, nasal and oral air flow, thoracic and abdominal respiratory effort, oxygen saturation, microphone and digitally time-synchronized videography. Sleep stages will be scored according to AASM criteria.

3.4 Echocardiography

Echocardiography will be performed in left supine position and includes measurement of left ventricular ejection fraction (LVEF), left ventricular enddiastolic and endsystolic diameter (LVEDD, LVESD) and volumina (LVEDV, LVESV), parameters of the right ventricular function (TAPSE, systolic PAP), size of the left atrium, diastolic left ventricular function, mitral regurgitation jet area (in cm^2) and parameters of dyssynchrony (APET, PPET).

3.5 Sample size estimation

The computed required sample size with following given parameters for two groups is as following: the estimated given effect size is 0,8 which means that the difference of the effect is relatively high.

This assumption is based on previous non-randomized studies showing that cardiac resynchronization therapy significantly reduces AHI in central sleep apnea: - 13.05 (CI -16.74 to -9.36; $p < 0.00001$) [13]. On the contrary, there is less effect on obstructive sleep apnea, which does not reach statistical significance: - 13.32 (CI -9.04 to 2.39; $p = 0.25$).

The alpha value is 0.05 and the power is 0.8 (beta = 0.2). The allocation ratio N2/N1 is 1 (equality). With these parameters the critical t value is 2.008 and the sample size in both groups is computed to be 26 patients. Therefore, the overall sample size is 52 patients. According to available literature [21-23], 65% of all screened patients will have a RDI ≥ 15. Therefore, the sample size should be 80 patients in order to get enough data to prove statistical significance for given parameters.

Computation results

t tests - Means: Difference between two independent means (two groups)

**Analysis:**	A priori: Compute required sample size		
**Input:**	Tail(s)	=	Two
	Effect size d	=	0,8
	α err prob	=	0,05
	Power (1-β err prob)	=	0,8
	Allocation ratio N2/N1	=	1
**Output:**	Noncentrality parameter δ	=	2,8844410
	Critical t	=	2,0085591
	Df	=	50
	Sample size group 1	=	26
	Sample size group 2	=	26
	Total sample size	=	52

This study will be conducted as a multicenter trial in three to five centers. It is planed to enroll 40-60 patients between 2014 und 2016 at the University Clinic in Innsbruck. External centers are expected to enroll 20-40 patients between 2014 und 2016.

4. REFERENCES

1. ESC Guidelines for the diagnosis and treatment of acute and chronic heart failure 2012. *Eur Heart J* 2012; 33:1787

2. Cleland JG, Daubert JC, Erdmann E, Freemantle N, Gras D, Kappenberger L, et al. The effect of cardiac resynchronization on morbidity and mortality in heart failure. *N Engl J Med* 2005; 352: 1539

3. Bristow MR, Saxon LA, Boehmer J, Krueger S, Kass DA, De Marco T, et al. Cardiac-resynchronization therapy with or without an implantable defibrillator in advanced chronic heart failure. *N Engl J Med* 2004; 350: 2140

4. Vardas PE, Auricchio A, Blanc JJ, Daubert JC, Drexler H, Ector H, et al. Guidelines for cardiac pacing and cardiac resynchronization therapy: The Task Force for Cardiac Pacing and Cardiac Resynchronization Therapy of the European Society of Cardiology. Developed in collaboration with the European Heart Rhythm Association. *Eur Heart J* 2007; 28: 2256

5. Dickstein K, Vardas PE, Auricchio A, Daubert JC, Linde C, McMurray J, et al. 2010 Focused Update of ESC guidelines on device therapy in heart failure. An update of the 2008 ESC guidelines for the diagnosis and treatment of acute and chronic heart failure and the 2007 ESC guidelines for cardiac and resynchronization therapy. *Eur Heart J* 2010; 31: 2677

6. Dickstein K, Bogale N, Priori S, Auricchio A, Cleland JG, Gitt A, et al. The European cardiac resynchronization therapy survey. *Eur Heart J* 2009; 30: 2450

7. Berger T, Zwick RH, Stuehlinger M, Dichtl W, Poelzl G, Edlinger M, Pachinger O, Hintringer F. Impact of Oxygen Uptake Efficiency Slope as a Marker of Cardiorespiratory Reserve on Response to Cardiac Resynchronization Therapy. *Clin Res Cardiol* 2011; 100: 159

8. Berger T, Pfeifer B, Hanser FF, Hintringer F, Fischer G, Netzer M, Trieb T, Stuehlinger M, Dichtl W, Baumgartner C, Pachinger O, Seger M. Single-Beat Noninvasive Imaging of Ventricular Endocardial and Epicardial Activation in Patients Undergoing CRT. *PLOS One* 2011; 6/1: e16255

9. Tang AS, Wells GA, Abraham WT, Ghio S, Hassager C, Goode G et al. Prevention of disease progression by cardiac resynchronization therapy for mild-to-moderate heart failure events. *N Engl J Med* 2010; 363: 2385

10. Bogale N, Witte K, Priori S, Cleleand JG, Aurricchio A, Gadler F, et al. The European Cardiac Resynchronisation Therapy Survey: comparison of outcomes between de novo cardiac resynchronisation therapy and upgrades (2011). *Eur J Heart Fail* 13:974

11. Curtis AB, et al. Biventricular pacing for atrioventricular block and systolic dysfunction (BLOCK-HF). *N Engl J Med* 2013; 368: 1585

12. Tremel F, Pépin JL, Veale D, Wuyam B, Siché JP, Mallion JM, Lévy P. High prevalence and persistence

of sleep apnea in patients referred for acute left ventricular failure and medically treated over 2 months. Eur Heart J 1999; 20: 1201

13. Lamba J, Simpson CS, Redfearn DP, Michael KA, Fitzpatrick M, Baranchuk A. Cardiac resynchronization therapy for the treatment of sleep apnoe: a meta-analysis. *Europace* 2011; 13: 1174

14. Wilkoff BL, Cook JR, Epstein AE, Greene HL, Hallstrom AP, Hsia H, Kutalek SP, Sharma A. Dual-chamber pacing or ventricular backup pacing in patients with an implantable defibrillator: the Dual Chamber and VVI Implantable Defibrillator (DAVID) Trial. *JAMA* 2002; 288: 3115

15. Young T, Finn L, Peppard PE, Szklo-Coxe M, Austin D, Nieto FJ, Stubbs R, Hla KM. Sleep disordered breathing and mortality: eighteen-year follow-up of the Wisconsin sleep cohort. *Sleep* 2008; 31: 1071

16. Grimm W, Sharkova J, Heitmann J, Jerrentrup A, Koehler U, Maisch B. Sleep-disordered breathing in recipients of implantable defibrillators. *PACE* 2009 ; 32 (suppl 1) : 8

17. Garrigue S, Pépin JL, Defaye P, Murgatroyd F, Poezevara Y, Clémenty J, Lévy P. High prevalence of sleep apnea in patients with long-term pacing : the European Multicenter Polysomnographic Study. Circulation 2007 ; 115 : 1703

18. Bitter T, Westerheide N, Prinz C, Hossain MS, Vogt J, Langer C, Horstkotte D, Oldenburg O. Cheyne-Stokes respiration and obstructive sleep apnea are independent risk factors for malignant ventricular arrhythmias requiring appropriate cardioverter-defibrillator therapies in patients with congestive heart failre. *Eur Heart J* 2011 ; 32 : 61

19. Chung ES, Leon AR, Tavazzi L, et al. Results from the Predictors of Response to CRT (PROSPECT) trial. *Circulation* 2008; 117: 2608

20. Shalaby, Atwood C, Hansen C, Konermann M, Jamnadas P, Lee K, Willems R, Hartley J, Stahmann J, Kwok J, Ni Q, Neuzner J. Feasibility of automated detection of advanced sleep disordered breathing utilizing an implantable pacemaker ventilation sensor. *PACE* 2006; 10: 1036

21. Sin DD, et al. Risk factors for central and obstructive sleep apnea in 450 men and women with congestive heart failure. *Am J Respir Crit Care Med* 1999; 160: 1101

22. Javaheri S, et al. Sleep apnea in 81 ambulatory male patients with stable heart failure: types and their prevalences, consequences, and presentations. *Circulation* 1998; 97: 2154

23. Bradley TD and Floras JS. Sleep apnea and heart failure Part I: Obstructive sleep apnea. *Circulation* 2003; 107: 1671
